# An Advanced Bioreactor Simulating Dynamic Physiological Conditions in the Human Ascending Colon: MimiCol^3^

**DOI:** 10.3390/pharmaceutics14051049

**Published:** 2022-05-13

**Authors:** Regine Beeck, Annemarie Dols, Felix Schneider, Dariah-Sohreh Seradj, Julius Krause, Philipp Schick, Werner Weitschies

**Affiliations:** Center of Drug Absorption and Transport, Institute of Pharmacy, University of Greifswald, Felix-Hausdorff-Str. 3, D-17489 Greifswald, Germany; regine.beeck@uni-greifswald.de (R.B.); anma.dols@t-online.de (A.D.); schneider-felix@mail.de (F.S.); dariah-sohreh.seradj@uni-greifswald.de (D.-S.S.); julius.krause@uni-greifswald.de (J.K.); philipp.schick@uni-greifswald.de (P.S.)

**Keywords:** sulfasalazine, MimiCol, dynamic colon model, in vitro metabolization, colonic microbiota

## Abstract

In recent years, the colon has become a hot topic in biopharmaceutical research as several in vitro models of the human colon have been presented. A major focus is on the characterization of the microbiota and its capabilities. The aim of the present study was to further develop the MimiCol, preserving its properties and accelerating data acquisition. Emphasis was placed on the simplicity of its design and easy scalability. To prove the viability of the concept, degradation of sulfasalazine was investigated, and the bacterial composition during the experiment was assessed by 16S rRNA sequencing. The transfer of the experimental conditions to the new model was successful. Commercially available components were implemented in the setup. The model MimiCol^3^ represented the colon ascendens satisfactorily in its properties regarding volume, pH value, and redox potential. 16S rRNA sequencing led to further insights into the bacterial composition in the vessels. Degradation of sulfasalazine was in good agreement with in vivo data. The new model of the colon ascendens MimiCol^3^ enabled us to collect more reliable data, as three experiments were conducted simultaneously under the same conditions.

## 1. Introduction

The composition of the human microbiome is as unique as a fingerprint [1]. Besides many other functionalities, it influences the degradation of drugs with consequences for the efficacy of therapy or unwanted side effects due to toxic metabolites [2]. The development of in vitro models is of immense importance in the investigation of the influence of the microbiome on new drugs and dosage forms [3]. These models must be appropriate for the specific physiological conditions of the colon and provide a reproducible method for testing drugs and dosage forms. A particular challenge in the development of these models is the representation of the gut microbiome. The microbiome in the gastrointestinal tract is composed of bacteria, fungi, and archaea as well as viruses with the highest bacterial density of 10^11^ to 10^12^ CFU/mL in the lower intestinal section of the colon, where anaerobic conditions prevail [4]. Enzymes such as ß-galactosidase, azoreductase, ß-xylosidase, nitroreductase, glycosidase, etc., produced and excreted by bacteria are particularly important for the metabolization of the drugs [5,6,7].

One of the first in vitro models depicting the colon was developed by Molly et al. in 1993, called “simulator of the human intestinal microbial ecosystem” (SHIME), which can simulate conditions in the gastrointestinal tract from the stomach to the distal part of the colon. It considers parameters such as enzyme and bile acid concentration, pH, temperature, nutrient supply, transit time and the anaerobic atmosphere [8]. Another model has been developed by the research group around Minekus and co-workers. They have designed two in vitro models called “TNO Gastro-Intestinal Model (TIM)”. The TIM-1 model represents the upper gastrointestinal tract including the stomach and small intestine, while TIM-2 represents conditions in the colon including characteristic motility patterns as well as water absorption [9,10]. Another model was created by Macfarlane et al. to simulate proximal, transverse, and distal colon conditions using human feces [11]. What all these models have in common is the complexity of their design and that it is time consuming to acquire enough data to show statistical significance [12,13]. Our first approach in the development of an in vitro model representing the dynamic conditions in the ascending colon was the MimiCol [14]. In brief, the model can simulate bacterial composition and dynamic pH levels in an anaerobic atmosphere mixed by slow moving perpendicular blades. A limitation to overcome is the fact that there is only one vessel, making it time consuming to produce statistically sound data.

The media used in the models developed so far are either buffers, which are only intended to reflect the correct pH value of the colon fluctuating between pH 5 and pH 8 (mean pH 6.5 ± 0.3) [15], or simulated colon media with the addition of specific bacterial enzymes such as azoreductase, galactomannase, β-glucosidase, dextranase, pectinase, or amylase that specifically degrade the polysaccharides used in the tested delivery systems [16,17]. Another option is the use of rat cecal contents with strict documentation of the feed composition for reproducible results and higher biorelevance of the media [18,19]. Human fecal slurries from healthy volunteers are also often used to investigate the influence of the human microbiome. Selected isolated bacterial strains such as *Bifidobacteria*, *Bacteroides* and *E. coli* are also used, again guaranteeing higher reproducibility in terms of the same composition of bacterial strains and comparable results between different laboratories [12,20]. It is of immense importance for a tool used in routine testing to achieve high reproducibility and, thus, high inter-day comparability. This can be achieved by using standardization, decreased complexity and process variables, and automated controls of critical process parameters.

In the determination of bacterial composition, 16S ribosomal RNA (rRNA) gene sequencing was shown to be the gold standard, as this method can be used to reliably analyze rare bacterial strains and bacteria that are difficult to cultivate in clinical practice [21]. 16S rRNA gene sequence analysis provided a suitable method for making both qualitative and quantitative statements about the bacterial strains present in the in vitro model. The 16S rRNA gene sequences have a length of about 1500 base pairs (bp) and consist of conserved uniform and nine hypervariable regions of varying conservation (V1–V9), the latter being used to distinguish bacterial species, family, or strains by matching against databases. The intestinal microbiota is mainly composed of representatives of five dominant phyla—*Bacteroidetes*, *Firmicutes*, *Actinobacteria*, *Proteobacteria*, and *Verrucomicrobia*—with the first two accounting for more than 90% of the bacteria [1]. In the proximal gut, the dominant groups are *Lactobacillus (Firmicutes)*, *Veillonella (Firmicutes)*, and *Helicobacter (Proteobacteria)*. In the duodenum, jejunum, and ileum, the most numerous groups are *Bacilli (Firmicutes)*, *Streptococcaceae (Firmicutes)*, and *Actinomycinaeae*, and in the colon, an increased proportion of *Lachnospiraceae (Firmicutes)* and *Bacteroidetes* was observed [22,23].

The aim of the present study was to further develop the MimiCol to enable simultaneous testing in up to three vessels while transferring the properties. This includes extension of the existing model to accelerate data acquisition. The focus was set on simplicity of design and easy scalability. Development included programming the control and monitoring of essential process parameters, considerations of the vessel design, the general structure of the model, planning of the experimental procedures and sample preparation. The new model was named MimiCol^3^. The subsequent experiments to observe the growth and composition of bacterial cultures were carried out to show the influence of shaking motion, pH value, temperature, and redox potential and to determine the bacterial strains by means of a 16S rRNA gene analysis. The aim was to adapt the process parameters and experimental procedure for optimized bacterial growth, which can subsequently be used to evaluate the bacterial metabolization of drugs. A reference experiment was conducted to show the degradation of sulfasalazine as a model drug as it is known that sulfasalazine is metabolized by bacterial azoreductases. The focus of these experiments was to investigate the relationship between bacterial growth and drug degradation and to demonstrate the biorelevance of the results obtained compared to in vivo data.

## 2. Materials and Methods

### 2.1. Materials

Azulfidine tablets (Pfizer, New York, NY, USA) with 500 mg of sulfasalazine were obtained from the university’s hospital pharmacy. Sulfasalazine at analytical standard was purchased from Fluka^®^ Analytical, Seelze, Germany. Schaedler broth was purchased as dry substance (Carl Roth, Karlsruhe, Germany) and reconstituted according to the manufacturer’s protocol. Sterile water for injection (Ph. Eur.) served as solvent in all cases.

### 2.2. Methods

#### 2.2.1. Dynamic Culture (MimiCol^3^)

**Figure 1:**
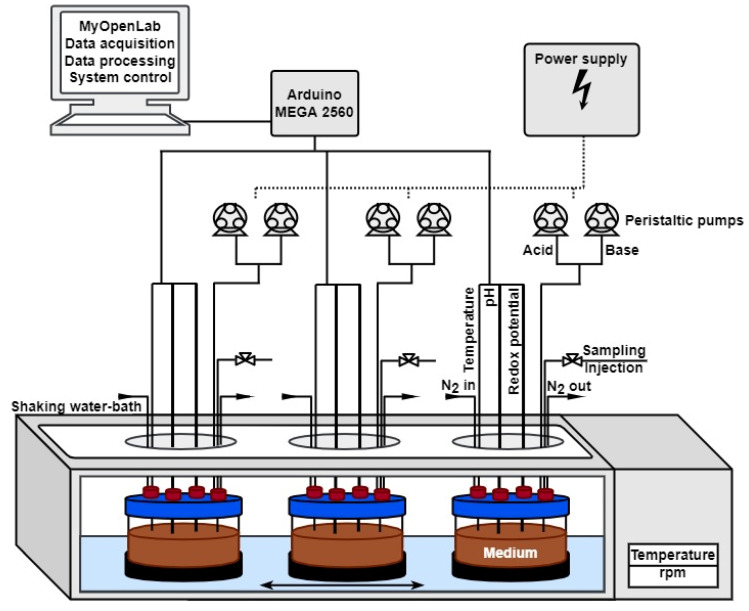
Schematic representation of the MimiCol^3^.

**Figure 2:**
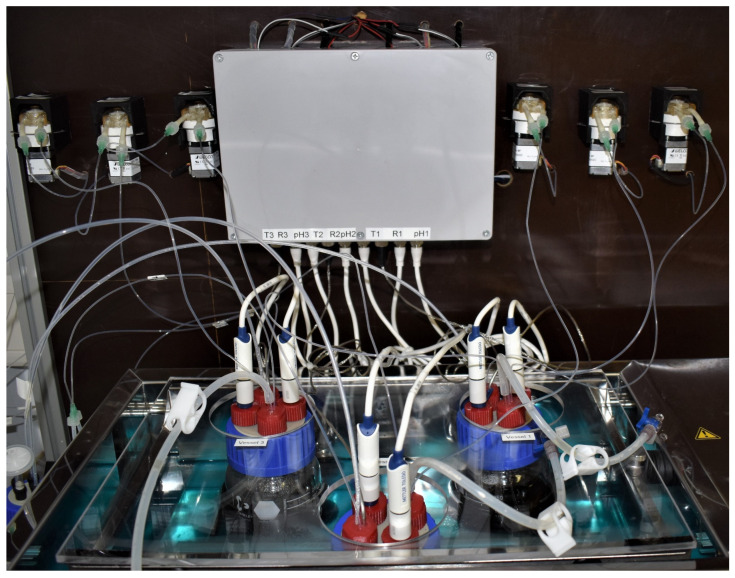
Picture of the assembled MimiCol^3^.

A schematic overview of the MimiCol^3^ is shown in Figure 1. Figure 2 shows a picture of the fully assembled device. Three 250 mL DURAN^®^ GLS 80^®^ wide mouth laboratory bottles with GLS 80^®^ thread (DWK Life Sciences GmbH, Mainz, Germany), with modified lids and access ports for electrodes, sample draw tubes, and tubing for pH control and gassing, are used as reactor vessels. The laboratory glass bottles are secured within the temperature-controlled shaking SW22 model water bath from the manufacturer JULABO GmbH (Seelbach, Germany) using a 3D-printed fixture. The shaking motion is set to 100 rpm and is intended to ensure homogeneous distribution and optimized bacterial growth.

The software MyOpenLab Version 3.11.0 (MyOpenLab^©^ 2017 Javier Velasquez, Chía, Republic of Colombia) is used to control the reactor. An Arduino MEGA 2560 (Arduino SA, Chiasso, Switzerland) microcontroller board serves as a communication platform between the different sensors and the peristaltic pumps. Each of the three vessels is equipped with the following sensors: a pH-electrode (InLab Expert, METTLER TOLEDO, Gießen, Germany) connected with Atlas Scientific’s EZO™-pH circuit; a redox-electrode (InLab Redox, METTLER TOLEDO, Gießen, Germany) connected with EZO™-ORP circuit; and a temperature sensor (Voltcraft^®^ Pt1000, Conrad Electronic AG, Hirschau, Germany) connected with an EZO™-RTD Circuit (AtlasScientific, New York, NY, USA). Furthermore, two peristaltic pumps (WPM1, WELCO Co., Tokyo, Japan) are installed for each vessel to conduct pH-regulation by pumping 1 M HCl and 1 M NaOH solution. A4988 Stepper Motor Driver Carriers (Pololu, Las Vegas, NV, USA) are used to control the pumps. The power supply is provided by a laboratory power pack (Digi 302-05, Mc Power Companies, Lee’s Summit, MO, USA).

The experimental conditions are based on those of the MimiCol [14] and displayed in Table 1. Bacteria are cultivated at a temperature of 37 °C under anaerobic conditions, which are generated via headspace gassing of the vessels with nitrogen. To guarantee comparable experimental conditions in all three vessels, the pH value, temperature, and redox potential are measured and displayed by the MyOpenLab program. It is also possible to set an automatic pH control with tolerable ranges (6.2 ± 0.25) for each vessel individually and to manually control each of the six pumps for individual acid and base supply.

A cryo-preserved complex microbiota derived from a batch cultivation process of a stool sample of a single healthy donor (female, age 25, no specific diet, no antibiotics intake) served as an inoculum for the experiments. The freshly collected feces was cultivated in a complex culture medium, derivedfrom Minekus et al. [10] in a bioreactor (Biostat^®^ Aplus, Sartorius, Göttingen, Germany) for 24 h. Afterwards, the broth was harvested in aliquots, spiked with glycerol, frozen at −20 °C and stored at −80 °C. The microbiota consisted of *Enterobacteria* (46%), *Clostridia* (25%), *Lactobacilli* (17%), *Bacteroides* (3%) and *Bifidobacteria* (9%). This was quantified by plating on selective agar media (Table 2).

#### 2.2.2. Experimental Procedure

After an initial inoculation with the cryo-preserved microbiota, the bacteria were incubated inside the bioreactor for 3 h. Manual media changes were performed at 3, 5 and 7 h to provide new energy sources to avoid substrate depletion and decline in bacterial colonies. The medium was exchanged in the ratio 90:10 with 15 mL of medium remaining inside the bioreactor and the addition of 135 mL Schaedler broth (pH 7.4). Schaedler broth contained 30 mg of powdered Azulfidine^®^ tablet (23.5 mg of sulfasalazine). The medium was only partially replaced so that the remaining bacteria could recolonize the fresh medium. Samples were withdrawn every 30 min to measure drug concentration (UV/VIS) and bacterial count (plating). To calculate the metabolization rate constants, the concentration in µg/mL was plotted against time in hours. The slope of the linear section of the curve was determined between sampling points 2 and 4 after each media change and converted to µM/h via the molar mass of sulfasalazine (398.38 g/mol).

To determine the drug concentrations during the culture experiments, samples were withdrawn at predetermined intervals and immediately centrifuged at 14,500 rpm for 6 min (MiniSpin^®^ plus, Eppendorf AG, Hamburg, Germany). The supernatant was diluted 10-fold with 0.1 M NaOH and analyzed photometrically at 450 nm in quartz cuvettes (path length = 1 cm) with a total volume of 2 mL (Cary 50, Varian, Inc., Palo Alto, CA, USA). Sulfasalazine showed a beneficial bathochromic absorption shift at pH values above pH 10, which reduced the background noise caused by various media components. Concentrations were calculated using a calibration curve of sulfasalazine analytical standard in 0.1 M NaOH.

To determine the optical density, samples were diluted 10-fold with distilled water and analyzed photometrically at 600 nm in quartz cuvettes (path length = 1 cm) with a total volume of 2 mL (Cary 50, Varian, Inc., Palo Alto, USA). A composition of 200 μL Schaedler broth and 1800 μL distilled water was used as the blank sample.

#### 2.2.3. Determination of Bacterial Count by 16S rRNA Sequencing

For the determination, the standard microbiota (date of sampling: 24 October 2017) and one sample each of the collected medium after five hours of experimental time from the vessels of the MimiCol^3^ were analyzed by external laboratories. For sample preparation, 50 mL of the standard microbiota was thawed, divided evenly among four falcon tubes, and centrifuged at 6000 rpm for 6 min. The bacterial pellets were resuspended in a small volume, combined and made up to 10 mL with supernatant. After a second cycle of centrifugation, the supernatant was poured off and the pellet was resuspended under the safety cabinet with 500 μL of the supernatant. This bacterial suspension could subsequently be transferred to a 1.5 mL Eppendorf tube. Samples from the media changes were prepared similarly. Here, however, only 25 mL of the sample was used, and the centrifuged pellets were resuspended with 300 μL supernatant in an Eppendorf tube. Samples were frozen at −20 °C and stored at −80 °C until transport. In brief, samples were collected in each vessel prior to media change at 5 h, stored in a tube containing stabilizing DNA buffer and then transported to the external laboratory. For the analysis underlying this work, two different operators were acquired. The company Eurofins Genomics (Ebersberg, Germany) was contracted and INVIEW Microbiome Profiling 3.0 was performed on a sample of the standard microbiota and a sample from the original MimiCol. This type of analysis is specialized for environmental samples, food samples, or samples of human origin and is therefore well suited to reliably detect bacterial strains in the samples. Next-generation sequencing was performed on the hypervariable regions V1–V3 as well as V3–V5 of the 16S rRNA, resulting in the taxonomic identification of the microbiome [27]. Furthermore, samples from the MimiCol^3^ were submitted to the University Medicine Greifswald. Sequencing was performed as described before in detail [28]. After DNA from samples was isolated (PSP Spin Stool DNA Kit; Stratec Biomedical AG, Birkenfeld, Germany), it was stored at −20 °C until analysis by 16S rRNA gene sequencing of the V1–V2 region on a MiSeq platform (Illumina, San Diego, CA, USA) [29].

## 3. Results

### 3.1. Process Parameters

**Figure 3:**
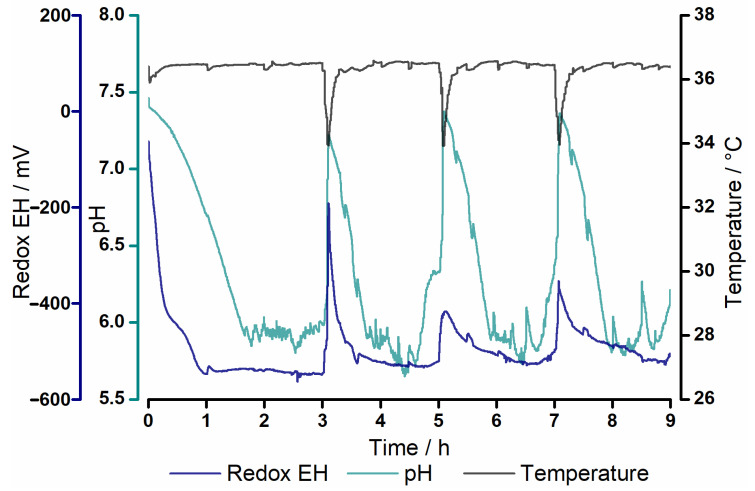
Process parameters at 100 rpm in the MimiCol^3^ over nine hours in 150 mL Schaedler broth at 37 °C and a pH of 6.2 ± 0.25 in vessel 1.

Figure 3 shows the process parameters in vessel 1 of the MimiCol^3^. The temperature dropped at 3, 5, and 7 h due to media change. Drifting of pH was allowed in a range of 6.2 ± 0.25. During media change, pH regulation was stopped and the pH increased due to the higher pH of the fresh medium (pH 7.4). The pH regulation was started again when pH fell below a level of pH 6.4. Redox potential was in the range of −503 ± 63 mV during most of the experiment. Only after the media changes was a decrease in redox potential noticeable.

### 3.2. Optical Density (OD_600_)

**Figure 4:**
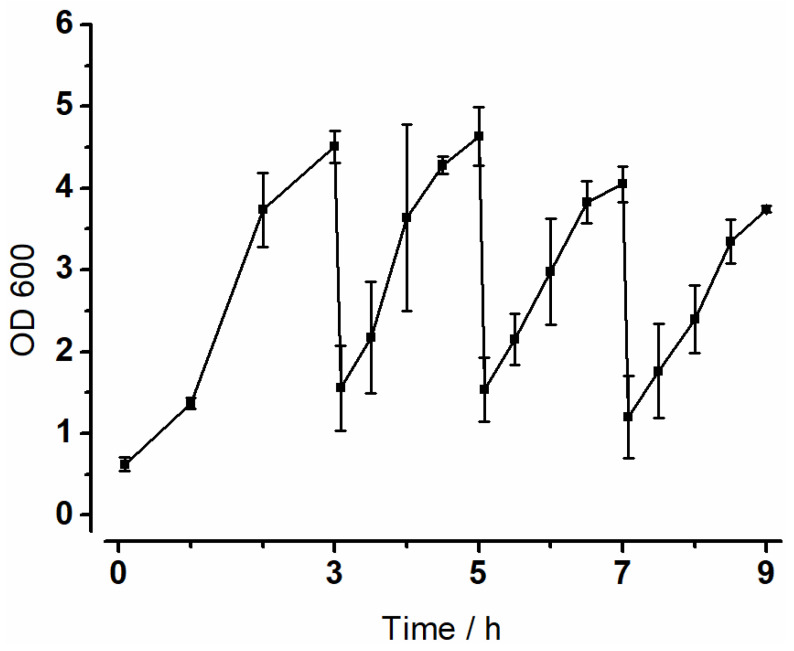
Optical density at 100 rpm in the MimiCol^3^ over nine hours in 150 mL Schaedler broth at 37 °C and a pH of 6.2 ± 0.25 (n = 3, mean ± SD).

To keep track of bacterial growth in the vessels during experiments, the optical density was measured. The results of the experiment with a shaking motion of 100 rpm are summarized in Figure 4 below. A distinct increase in absorption was visible in all vessels. In the first interval, a lag-phase of one hour was observed. Overall, the individual graphs showed similar progressions and both minimum and maximum values of the absorption differed only slightly. After the first media change, there was a renewed increase in absorption, but this stagnated in all three vessels between 4.5 and 5 h. This phenomenon was observed again after 6.5 h. Growth was lower in the last interval than in the first three intervals.

### 3.3. 16S rRNA Sequencing

**Figure 5:**
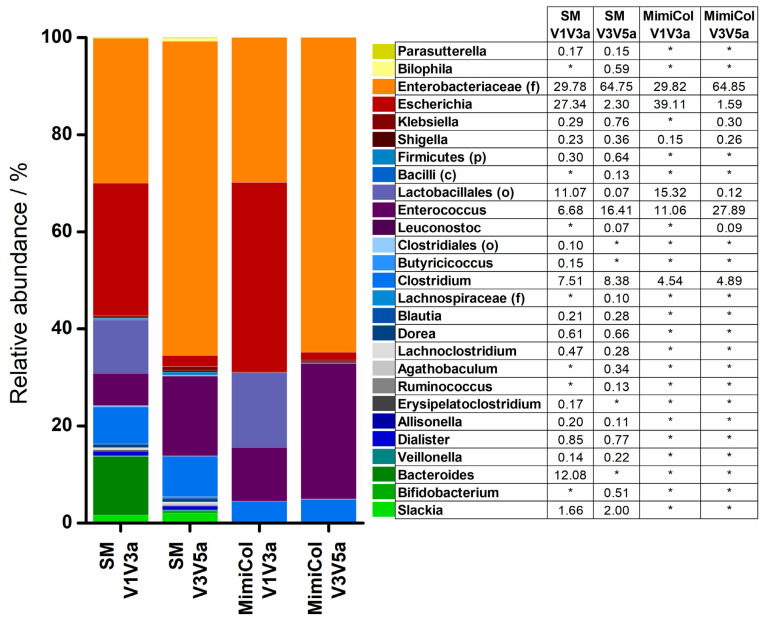
16S rRNA sequencing results of a sample of standard microbiota (SM) and MimiCol with focus on hypervariable regions V1–V3a and V3–V5a (* = not detected).

**Figure 6:**
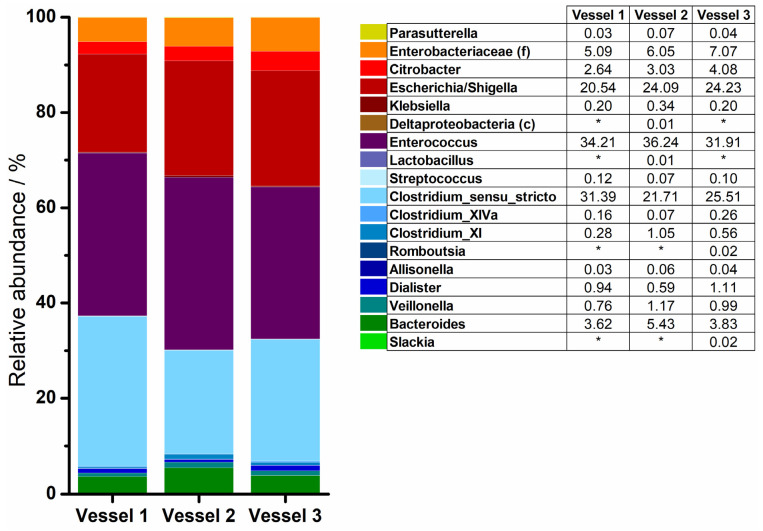
16S rRNA sequencing results of a sample from MimiCol^3^ with a focus on hypervariable regions V1–V2 (* = not detected).

Various samples were examined in a phylogenetic analysis of the 16S rRNA sequence. Bacterial genera are represented in Figure 5 and Figure 6. In some cases, where a more precise characterization was not possible, other taxonomic ranks were presented instead and marked as follows: (p)—phylum, (c)—class, (o)—order, (f)—family. Bacteria of the four different phyla—Actinobacteria, Bacteroidetes, Firmicutes, and Proteobacteria—were found in the samples. The composition of the standard microbiota is shown on the left in Figure 5. Analysis of the hypervariable regions V1–V3 led to a higher diversity of identified genera than analysis of regions V3–V5. The largest share was accounted for by the *Proteobacteria* especially the *Enterobacteriaceae*, but most of the identified genera belonged to the phylum *Firmicutes*. *Bacteroidetes* were only represented by *Bacteroides*. The relative abundance of *Actinobacteria* was the lowest in comparison to the other phyla.

Changes of varying magnitude were observed in the composition of the standard microbiota in both models after five hours. The bacterial diversity was lower in MimiCol and in MimiCol^3^. In MimiCol, the general composition of the bacterial strains remained similar and is shown on the right hand side in Figure 5. Thus, the *Proteobacteria* including the *Enterobacteriaceae* took the largest share, followed by the *Firmicutes* phylum with the *Lactobacillales* as the predominant order and the *Clostridia* species. The phylum of *Actinobacteria* with *Corio-* and *Bifidobacteria* species previously determined in the standard microbiota was no longer detectable in the MimiCol after five hours of testing. The exact same was the case for the *Bacteroides*.

Results of the sequencing of the experiment in the MimiCol^3^ are summarized in Figure 6. Hypervariable regions V1–V2 were examined. The bacterial composition inside the three vessels differed slightly. According to this, the phylum of *Firmicutes* with 63.13 ± 4.13% took the largest share of the sample taken after five hours with *Enterococcus* and *Clostridium* species as the dominant bacterial order. The phylum of *Proteobacteria* was determined with 32.57 ± 3.67% and was dominated by *Escherichia/Shigella* species. *Bacteroidetes* accounted for 4.29 ± 0.99%. *Actinobacteria* showed the lowest abundance in the samples.

### 3.4. Degradation of Sulfasalazine

**Figure 7:**
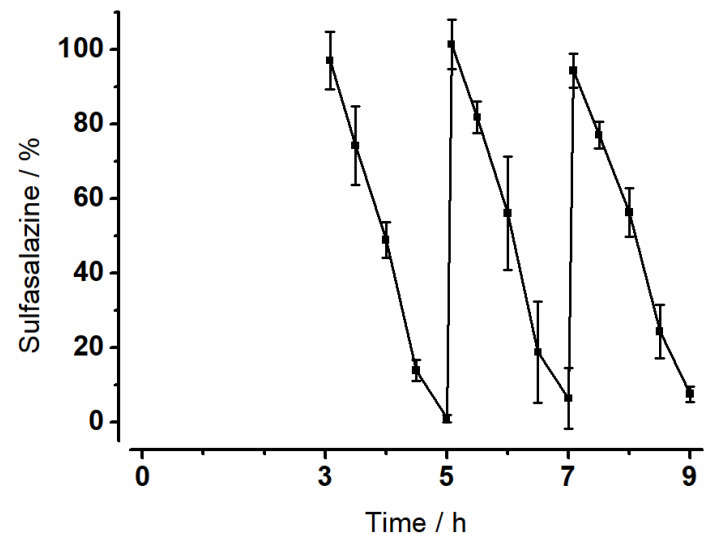
Metabolization of sulfasalazine in MimiCol^3^ in addition to the reactor after a media change at 3 h, 5 h, 7 h after inoculation (150 mL Schaedler broth, 37 °C, pH 6.2 ± 0.25, 100 rpm, n = 3, mean ± SD).

The metabolization of sulfasalazine was investigated in the MimiCol^3^. Figure 7 shows the degradation of sulfasalazine over time. Sulfasalazine was added after media change at 3, 5 and 7 h. The amount of sulfasalazine in the vessels was calculated using a calibration curve. An almost linear decrease was observed. The degradation became slightly slower towards the end of the intervals. The amount of remaining sulfasalazine at 5, 7 and 9 h increased over time.

**Figure 8:**
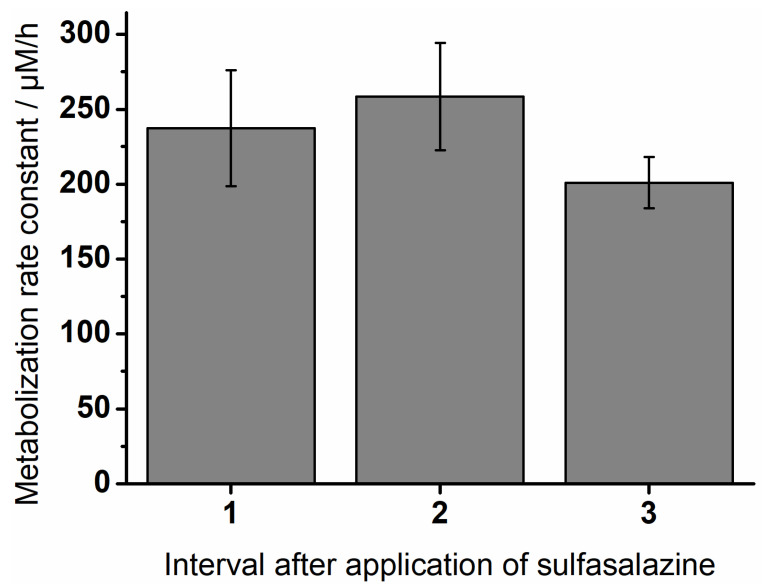
Metabolization rate constants of sulfasalazine in MimiCol^3^ (n = 3, mean ± SD).

To compare the curves, the metabolization rate constants for the individual intervals were calculated. They are shown in Figure 8. The highest rates occurred in the second interval between five and seven hours after inoculation. It decreased towards the end of the experiment.

## 4. Discussion

With the increased interest in colonic microbiota, the need for in vitro models has increased in recent years. The current solutions are overly complex. We have successfully addressed this with the MimiCol bioreactor [14]. However, the reactor had to be developed further to facilitate replication.

Therefore, a comparison with data obtained in MimiCol in previous experiments is essential to show the benefits of the new model and the successful method transfer to the new model [14]. Process parameters were comparable in both models. However, it was noticeable that the redox potential was higher in MimiCol^3^ and closer to physiological conditions. Mean redox potential in the ascending colon is −415 mV [30].

Optical density (OD_600_) was used to monitor bacterial growth. Aiming for an exponential growth of bacteria, the medium was changed at 3, 5 and 7 h. This maintained the nutrient supply at an elevated level and removed metabolites. Improving the experimental procedure, the first growth phase was extended to 3 h to achieve a more uniform growth of bacteria. Compared to experiments in MimiCol shown in previous work, this led to higher and more uniform absorptions in the individual intervals.

In addition to optical density for quantification of the bacteria, determination of the bacterial composition was conducted by 16S rRNA gene sequencing. In previous experiments with the MimiCol, selective agar media were used for the closer characterization of the bacterial strains. Plating is a suitable and fast method to obtain a general overview of the bacterial strains in a sample. Agar media were chosen to represent genera from the four dominant phyla. Cultivated *Enterobacteria* represent the phylum *Proteobacteria*. *Clostridia* and *Lactobacilli* represent the phylum *Firmicutes*. *Bacteroides* belong to the *Bacteroidetes* and *Bifidobacteria* to the *Actinobacteria*. Plating is easily performed in the laboratory and only limited by the number of different media existing. Characterization is only possible up to a certain genus and not up to a certain species. To overcome this limitation, 16S rRNA sequencing was performed in addition. The method is more precise, gives more information, but is expensive, cannot be performed on-site and takes more time than plating. The analysis of the standard microbiota by 16S rRNA gene analysis showed a different composition of the bacterial strains than the microbiota composition mentioned as an example in the introduction. According to Yang et al., the phylum *Bacteroidetes* occupied a share of 30%, whereas in the analyzed standard microbiota, this strain was only represented with 12% [23]. When plated, only 3% of the standard microbiota were *Bacteroides*. During sampling, contact with oxygen could not be avoided. As such, the viability of the obligate anaerobic *Bacteroides* might be compromised. This could influence the abundance of *Bacteroides* found with different methods as, with plating, only the viable bacteria can be cultured, while with 16S rRNA sequencing, non-viable bacteria are also recorded. The main part of the standard microbiota was dominated by the *Proteobacteria* phylum. Plating only detected *Enterobacteria*, whereas sequencing also detected other *Proteobacteria*; thus, relative abundance in sequencing results was higher. Compared to the sequencing results, more *Firmicutes* were detected by plating. RCM is a non-selective growth medium which promotes the growth of several anaerobic and facultative-anaerobic Gram-negative bacteria under anaerobic conditions. MRS medium is apt for the growth of lactic acid bacteria, including *Lactobacillus, Streptococcus, Pediococcus* and *Leuconostoc* [31]. The phylum *Actinobacteria* had lower abundances in sequencing than by plating. In general, the diversity and individuality of the composition of the donors’ gut microbiota could be a reason for differences in the composition of the standard microbiota. Likewise, some bacterial species may have been lost during cultivation. Oxygen contact during sampling and further processing could have led to a loss of obligate anaerobic strains. Overall, plating is sufficient for a general overview of the composition of the culture; sequencing provides more details. Regarding the composition of the standard microbiota, it must also be considered that the bacteria were obtained from a stool sample. According to Moore et al., rapid growth of the bacteria occurs in the cecum and ascending colon and, afterwards, composition remains constant, which leads to the conclusion that bacteria of the transverse and descending colon and rectum are at the maximum stationary phase of growth and that the bacteria in feces represent the flora of the colon [32]. Marteau et al. contradict this statement. They state that feces cannot directly reflect the microbiota found alive in the intestine and, in addition to living microorganisms, also contain already dead bacteria that cannot be cultivated [33].

The basic composition of the standard microbiota in *Proteobacteria*, *Firmicutes* and other phyla changed only slightly during the experiments in both models. Only the phylum of *Actinobacteria* could not be detected in the experiments, in contrast to the analysis of the standard microbiota. The representatives of this phylum can be facultative to strictly anaerobic. In both experiments, *Enterobacteria* were recorded as the largest proportion of the sample, followed by *Lactobacilli* and *Clostridia* species, whereby the proportion of *Enterobacteria* was greater in the MimiCol^3^ and *Lactobacilli* were less represented. It must be pointed out that *Bacteroides* could be detected in the new model in contrast to the MimiCol.

The examined and cultivated anaerobic bacterial strains in this experiment showed distinctive characteristics regarding oxygen tolerance. The genera *Enterobacteria* and *Bifidobacteria* are facultative anaerobic microorganisms, while *Bacteroides* and *Clostridia* were obligate anaerobic. The genus *Lactobacilli* also belongs to the anaerobic bacteria but could survive as an aerotolerant species under an oxygen-containing atmosphere [34]. Since representatives with different oxygen tolerance were present in the samples, it can be assumed that both models guaranteed good conditions for the growth of these bacterial species regarding the representation of the anaerobic atmosphere in the colon. In addition to the anaerobic atmosphere, the cultivation medium also has a major influence on the growth of the bacteria. Schaedler broth was chosen as a liquid medium rich in nutrients, in which a large number of anaerobic organisms grow abundantly. The medium is based on CASO broth, with the addition of L-Cystin (reducing agent) and hemin, among others, for optimization of anaerobic growth [35].

The investigations on bacterial growth showed that relevant bacterial species of the colon could be cultivated in both models and the factors influencing bacterial growth were characterized in more detail. These observations formed the basis for the subsequent investigations of the enzymatic degradation of the drug sulfasalazine. Cultivated bacterial strains were able to show that they were capable of successfully enzymatically degrading the drug sulfasalazine. A constant degradation of sulfasalazine was recorded in the MimiCol^3^, which was comparable in all three vessels. Towards the end of each interval, metabolization slowed down slightly. This observation could be explained by the decreasing nutrient supply, which limited bacterial growth and, thus, enzymatic activity. Moreover, the calculated metabolization constants were comparable to the values in the MimiCol [14]. Sulfasalazine is degraded by azoreductases to the active ingredient mesalazine and the metabolite sulfapyridine [36]. Mesalazine is considered an effective drug in the treatment of, for example, chronic inflammatory bowel diseases, while sulfapyridine is used in rheumatoid arthritis and is responsible for side effects such as agranulocytosis, gastrointestinal symptoms, and skin reactions. Depending on the dosage form, at least 25% of the active ingredient mesalazine is absorbed, while the degradation product sulfapyridine is completely absorbed into systemic circulation [37,38]. Measuring the concentration of sulfapyridine in blood plasma after oral sulfasalazine administration has already been used as a marker for orocecal transit time [39,40,41]. Therefore, the appearance of sulfapyridine in blood plasma after sulfasalazine administration is a good indicator of sulfasalazine degradation and was investigated in vivo by a study by Kellow et al. [42]. When administered directly into the cecum, the first sulfapyridine concentrations of 0.1 to 0.6 µg/mL were measured in the plasma after one to six minutes. This rapid formation of sulfapyridine in vivo is in excellent agreement with the fast degradation of sulfasalazine observed using MimiCol^3^. The measured plasma profiles showed that a complete degradation of the sulfasalazine took place after about 130 min. In the MimiCol^3^, an almost complete degradation could be shown in the period of 120 min, which is also in good agreement with the in vivo observation. Most of the azoreductase-producing bacteria belong to the genera *Clostridium* and *Eubacterium* [43,44]. This coincidence between the degradation time of sulfasalazine in the in vitro model MimiCol^3^ and the flooding of sulfapyridine in blood plasma indicates that sufficient bacteria were cultured to represent the in vivo conditions in the colon with respect to sulfasalazine degradation.

In contrast to previously described in vitro models, the model MimiCol^3^ developed in this work was able to represent the colon ascendens satisfactorily in its characteristic properties regarding volume, pH value, and redox potential. In the MimiCol^3^, the experimental conditions were kept simple, and the experimental time was limited to as few hours as possible, although the length of the experimental time can also be adapted to the experimental objective. The advantages of the extended model are mainly that three vessels were used in parallel. In contrast to the previous work with the MimiCol model, the vessel as well as all parts of the lid design can be sterilized and exchanged easily. Control of the individual vessels nevertheless remained independent of each other. Data were obtained under the exact same conditions on the same day. Due to the automatic pH control, it was possible to display dynamic pH profiles. Use of a shaking water bath made the blades used for mixing in the MimiCol obsolete. In summary, MimiCol^3^ provides higher throughput, allowing for screening of different drugs and xenobiotics with improved efficacy.

Compared to the existing in vitro models SHIME and TIM-2, which are also designed to simulate the physiological conditions of the colon, the MimiCol^3^ has a simplified design. In addition, the experimental time could be significantly shortened by using a standard microbiota, whereas in the SHIME and TIM-2, a fresh feces sample first had to be pre-cultured for several hours. The standard microbiota had the additional advantage that reproducible results were achieved in the previous version and in MimiCol^3^. The volume of 150 mL remained unchanged, adapted to the physiological volumes in the colon ascendens. Volume in the SHIME is significantly higher with 1000 mL for the ascendens compartment. Dynamic conditions as introduced in the previous work on the MimiCol could also be represented in the MimiCol^3^.

## 5. Conclusions

In summary, constructing an in vitro model to simulate conditions in the ascending colon was successful, including the aim of a simple setup and simultaneous testing in several vessels. Cultivating the standardized microbiota was successfully conducted in the MimiCol^3^. Further information on the composition of the bacterial cultures was obtained via 16S rRNA sequencing. With these additional adaptations of the model and the experimental procedure, the MimiCol^3^ can represent a valuable tool to investigate the influence of our gut microbiome on the degradation of drugs and dosage forms.

## Figures and Tables

**Table 1:** Process parameters in the in vitro model MimiCol³ compared to in vivo conditions in the colon.

	In Vivo [17]	MimiCol^3^
**Volume**	170 ± 40 mL ascending colon [24]	150 mL
**Motility**	Pendulum movements, segmentations and fast mass transfer	Shaking at 100 rpm
**pH**	5–8 (mean pH 6.5 ± 0.3) [25]	6.2 ± 0.25 pH profile starting at pH 7.4
**Microbiota**	10^11^ CFU/mL [26]	Standard microbiota derived from a fecal sample of a healthy human volunteer (2.82 × 10^10^ CFU)
**Temperature**	37 °C	37 °C
**Anaerobic atmosphere**	Anaerobic, microaerophilic	Head space gassing with N_2_

**Table 2:** List of agar media used for determination of colony forming units of bacterial sub-populations.

Bacterial Sub-Population	Agar Medium
Enterobacteria	MacConkey agar
Lactobacilli	deMan, Rogosa and Sharpe (MRS) agar
Bifidobacteria	Bifidus Selective Medium (BSM) agar
Clostridia	Modified reinforced clostridial medium
Bacteroides	Modified Schaedler agar

## Data Availability

The data presented in this study are available at the University of Greifswald storage system.
